# A High Isolation Series-Shunt RF MEMS Switch

**DOI:** 10.3390/s90604455

**Published:** 2009-06-05

**Authors:** Yuan-Wei Yu, Jian Zhu, Shi-Xing Jia, Yi Shi

**Affiliations:** 1 Department of Physics, Nanjing University, Nanjing, 210093, China; E-Mail: yshi@nju.edu.cn; 2 National Key Lab. of Monolithic Integrated Circuits and Modules, Nanjing, 210016, China; 3 Nanjing Electronic Devices Institute, Nanjing, China; E-Mails: zhujianyiya@yahoo.com.cn (J.Z); mems@nedi.cn (S.X.J)

**Keywords:** series-shunt, RF MEMS switch, metal-contact, electrical model

## Abstract

This paper presents a wide band compact high isolation microelectromechanical systems (MEMS) switch implemented on a coplanar waveguide (CPW) with three ohmic switch cells, which is based on the series-shunt switch design. The ohmic switch shows a low intrinsic loss of 0.1 dB and an isolation of 24.8 dB at 6 GHz. The measured average pull-in voltage is 28 V and switching time is 47 μs. In order to shorten design period of the high isolation switch, a structure-based small-signal model for the 3-port ohmic MEMS switch is developed and parameters are extracted from the measured results. Then a high isolation switch has been developed where each 3-port ohmic MEMS switch is closely located. The agreement of the measured and modeled radio frequency (RF) performance demonstrates the validity of the electrical equivalent model. Measurements of the series-shunt switch indicate an outstanding isolation of more than 40 dB and a low insertion loss of 0.35 dB from DC to 12 GHz with total chip size of 1 mm × 1.2 mm.

## 1. Introduction

Switches are one of the essential components in RF circuits and systems. An RF MEMS switch has the following advantages: low loss, high isolation, good linearity, broad band and low power consumption [1]. Recently, many MEMS series switches have been studied [2-7]; they showed significantly wider bandwidth compared with capacitive switches, allowing them to be used in circuits intended for multiple frequency bands, but some applications, such as mobile communication systems, WLANs, electronically steerable antennas (ESA), and automatic test equipment, demand RF switches with both low insertion loss and very high isolation performance [2]. If the isolation characteristics of switches are lower, then more intermodulation is produced caused by leakage of signals from another channel, thus the RF system performances will be debased [5]. Several methods have been used in the design of high-isolation switches. Metal-contact series MEMS relays [5-6], using composite bridges structures with the smaller contact areas and a much broader signal line gap, achieve a good isolation from DC to 40 GHz, but the process is relatively complex, and the isolation cannot reach up to 40 dB usually up to X/Ku band. Capacitive shunt MEMS switches obtain a high isolation using inductive tuning or multi-bridge tuning at X/Ku/Ka band [8], but it is not suitable for wide band applications. Another way is the series/shunt configuration which is commonly used in wideband PIN-diodes or FET switches, and also can be integrated by MEMS technology [9], for example, absorptive MEMS switches [10], capacitive series-shunt switches [11] and DC-contact series-shunt switches [12-13]. For increasing the isolation, 1 series and 2 shunt switch structures are used, while shunt switches separated by a quarter wavelength section of transmission line [13]. This series-shunt switch suited for DC-20 GHz applications, however, is not compact.

In this paper, a series-shunt MEMS switch with a very high isolation characteristic and compact size is presented. To avoid fabrication complexity, three identcal 3-port metal-contact MEMS switches are employed. Furthermore, in order to evaluate and optimize RF performances of the series-shunt switch, a small-signal electrical model is extracted from measured results of 10 metal-contact MEMS switches.

## 2. 3-Port MEMS Switch

**Figure 1.**
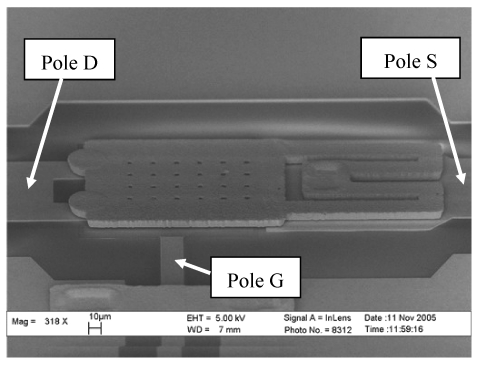
SEM image of 3-port ohmic MEMS switch.

MEMS switches are core components for implementing the series-shunt switch. 3-Port metal-to-metal cantilever series in-line switches [7] are employed due to their good performance and compact size. The SEM observation of the RF MEMS switch is shown in Figure 1. It contains CPW transmission lines, metal cantilever, anchor and “double U-shape” beam. The area of switching part is 340 μm × 160 μm. When a drive voltage is applied between the cantilever and the bottom electrode (pole Gate), the cantilever pulls down until the dimples and it makes the RF pole S (Source) connect with pole D (Drain). A 3-port structure just like that existing in an FET switch is developed to eliminate the influence on the RF signals caused by the DC drive signals.

Electro-mechanical simulations and optimizations of the cantilever structure were performed using Coventorware software. The pull-in voltage was simulated and shown in Table 1 by assuming that the cantilever beam doesn't contain thin-film stress. Knowing the mechanical resonant frequency *f_0_* and the effective mass *m* of the cantilever from the simulated results, the spring constant *k* is determined by Equation (1):

(1) $$\varpi_{0}=2\pi f_{0}=\sqrt{\frac{k}{m}}\Rightarrow k={\left(2\pi f_{0}\right)}^{2}•m$$

Assuming the damping coefficient of the cantilever is relatively small, the calculated switching time is given by [1]:

(2) $$t_{s}=3.67\frac{V_{P}}{V_{S}\varpi_{0}}=0.584\frac{V_{P}}{V_{S}f_{0}}$$

where *Vp* is the pull-in voltage and *Vs* is the drive voltage. For fast switching, *Vs* is adopted to 1.4*Vp* usually. Table 1 gives the design parameters of the switch structure.

The average pull-in voltage is 28 V, which is measured on several switches of a 4 inch wafer. For fixed-fixed beams or membrane bridges, the residual stress results in not only increases of the gap, but also higher spring constant [14]. But for cantilever beam, it is not fixed at one end, thus the residual stress within the beam is released and the spring constant is independent of the stress. However the stress gradient induced the beam an upward deflection which can be observed under the microscope, and the pull-in voltage is proportional to the $g_{0}^{3/2}$ (gap between the beam and bottom Gate electrode), if a deflection of 0.82 μm is assumed, then the simulated pull-in voltage agrees well with the measurements.

**Figure 2.**
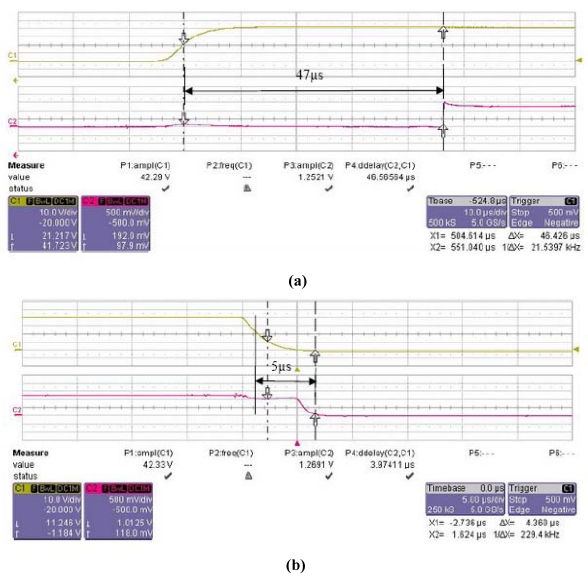
Measurements of switching on/off time: (a) at rising and (b) falling actuations.

To measure the switching time, a square wave of 0/40 V with a frequency of 1 KHz is biased at the pole G to control the switch state. A voltage of 2.5 V is in series with a 10 KΩ resistance to pole S and another 10 KΩ resistance is in series with pole D to ground. Thus the switch time can be measured by detecting the voltage wave at pole D using a LeCroy 6051 A oscilloscope in different switch states controlled by bias voltage. Figure 2 shows the curves of the measured switching times. The measured switching on/off time is 47 μs at rising and 5 μs at falling, which also closely matches the calculated value from Table 1.

**Figure 3.**
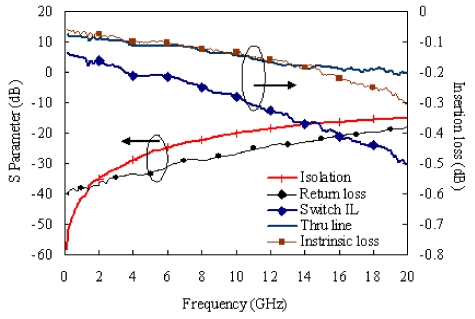
Performance of MEMS switches.

RF performances of 10 RF MEMS switches have been measured with an on-wafer test instrument. The measured results are shown in Figure 3. The average insertion loss is less than 0.5 dB between the input port and out port in a frequency range of DC to 20 GHz, and the intrinsic loss of the MEMS switch is similar as the RF through calibration line with a length of 700 μm.

## 3. Series-shunt MEMS Switch

**Figure 4.**
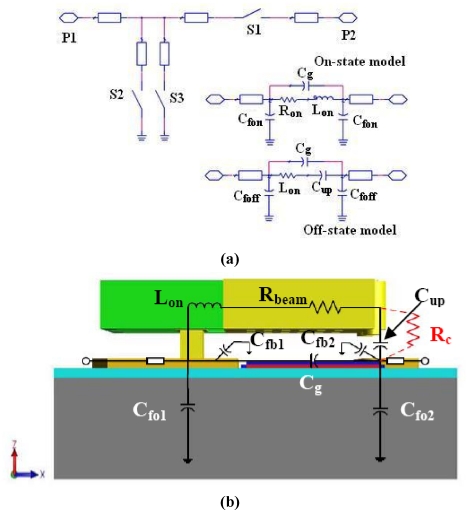
(a) Equivalent circuit of the series-shunt switch; (b) The model of a 3-port MEMS switch.

The high isolation switch using 3-port ohmic MEMS switches has been employed for many circuit applications. The schematic diagram is shown in Figure 4. Totally 1 series and 2 shunt switch structures are used. When the series one is closed and the shunt two are open, the series-shunt RF MEMS switch is at ON state. On the other hand, the series one is open and the shunt two are closed. Therefore, it is at OFF state. In the design procedure, circuit model is used for optimization so that the design period could be shortened.

**Figure 5.**
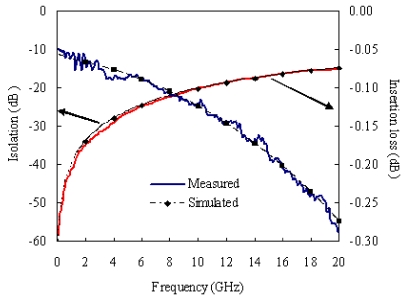
Comparison of the measured and modeled parameters of 3-port MEMS switches.

Figure 4(b) shows the structure-based small-signal models for the 3-port ohmic MEMS switch in detail. *C_g_* refers to the signal-line coupling capacitance due to the gap between each broken signal line and *C_up_* refers only to the off-state capacitance due to the distance of the overlapping part between the front end of the signal line and the dimple areas in the cantilever. *R_on_* is made up of DC-contact resistances *R_c_* and a short section of beam resistance *R_beam_*. *L_on_* represents a series inductance of the beam. The narrow “double U-shape” beam shows relatively large inductance values which influence the RF performance so much as off-state. For predicting accurate model, the fringing capacitances (*C_fon_* and *C_foff_*) can't be ignored. The off-state *C_foff_* contains signal line fringing capacitances (*C_fo1_*, *C_fo2_*) and off-state beam effect fringing capacitances (*C_fb1_*, *C_fb2_*). Similarly, the on-state *C_fon_* expresses the on-state beam effect (*C_fbon1_*, *C_fbon2_*) and capacitances (*C_fo1_*, *C_fo2_*). The different switch bias mode will influence the beam effect fringing capacitances [5,15] due to the beam's movement relative to single lines. Here to evaluate the RF performances of the 3-port MEMS switch, we just fit *C_fon_* and *C_foff_* instead of calculating each capacitance mentioned above. To extract the parameters related to the small-signal model, an RF through test pattern was additionally designed as well as an RF open test pattern. By removing the loss of the calibration line, the result is less than 0.3 dB and the best is 0.1 dB at 6 GHz. The isolation of the switch is 24.8 dB. Using small-signal model of switch together with the measured results, extracted parameters of the switch are shown in Table 2. Figure 5 shows the measured and modeled S-parameters for 3-port ohmic MEMS switches in the on-state and the off-state. The excellent agreement demonstrates that the proposed electrical model can be used for designing a MEMS switch based circuit.

Using the small-signal model, first, a MEMS switch with one series and one shunt switch structures is designed and the simulated isolation is only 30.2 dB at 10 GHz. It doesn't meet the high-isolation channel circuits' demands. Thus a high-isolation series-shunt switch requires one series and two shunt switches and the configuration has been optimized with the following three approaches. One is an absorptive switch. The second is a series-shunt switch, with two shunt switches separated by a quarter wavelength section of transmission line. The third is also a series-shunt switch, while 2 shunt switches closed on the two sides of transmission line. Finally the simulated isolation is 40.1, 40.9 and 44.1 dB at 10 GHz, respectively. But the front two kinds of switch configuration need transmission lines at the input port, output port, and the section among the two shunt switches to take the impedance matching into account, and the total length of signal lines is about 7 mm. The last one structure is more compact because each MEMS switch is located as close as possible, so the area of the series-shunt switch has been optimized to 1 mm×1.2 mm.

## 4. Fabrication and Results

**Figure 6.**
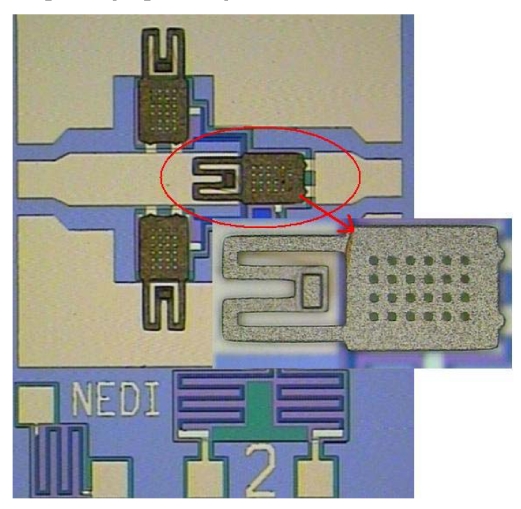
Microphotograph image of the fabricated series-shunt switch.

The fabrication process of the series-shunt MEMS switch can be compatible with the process of a single 3-port MEMS switch. The low temperature surface sacrifice layer technique was applied. First, a layer of silicon dioxide (SiO_2_) was thermally grown to insulate the switch from the silicon substrate. Second, Si-metal bias line (1,000 Å) and bottom electrodes (3,000 Å) were patterned and silicon nitride (Si_3_N_4_, 1,000 Å) was deposited using plasma-enhanced chemical vapor deposition (PECVD). A layer of PMMA (2 μm) was then spin-coated and cured as a sacrificial layer. The PMMA was two steps etched to define the dimples (5,000 Å) and the anchor. Au (3,000 Å) was sputtered to realize contact metal, which was also as a seed layer, and a thick cantilever beam (6 μm) was electroplated. Finally, the sacrificial layer was removed using O_2_ reactive ion etching (RIE) process. Figure 6 shows the microphotograph image of the fabricated series-shunt MEMS switch.

**Figure 7.**
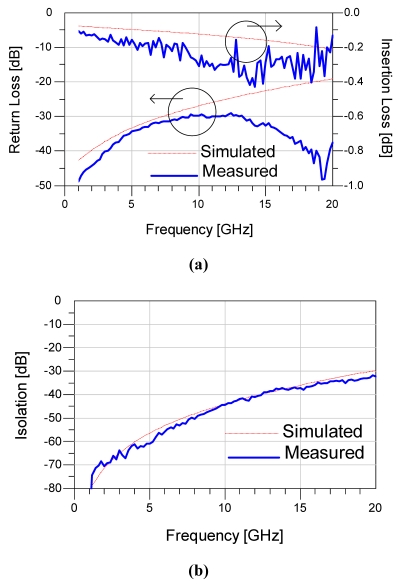
Measured and modeled S-parameters for the series-shunt switch, (a) On state; (b) Off state.

The measured pull-down voltage was 20 - 40 V for several series-shunt switches. RF responses of the series-shunt switch performed on a 8510C vector network analyzer and cascade RF probe station using a SOLT calibration in lab ambient. Figure 7(a) shows the measured and simulated results for the switch in the ON state (series-switch in down state while 2 shunt switches in up state). The measured insertion loss is 0.18, 0.30 and 0.40 dB at 6, 12 and 18 GHz while the return loss is better than 29 dB up to 20 GHz. When series-switch is in an up state while 2 shunt switches are in down state, the series-shunt switch is OFF state. Thus the measured isolation is 56.2, 41.1 and 32.2 dB at 6, 12 and 20 GHz, respectively. The simulation results of the circuit model shows a good agreement with the measurement results, as shown in Figure 7. The measured data shows an outstanding isolation of more than 40 dB and a low insertion loss of 0.35 dB from DC to 12 GHz.

## 5. Conclusions

A novel structure for a series-shunt switch using electrostatically actuated 3-port ohmic MEMS switches has been fabricated. The series-shunt RF MEMS switch exhibits very high isolation (> 40 dB), low loss (< 0.35 dB) and good matching up to 12 GHz. The structure-based small-signal model for the 3-port ohmic MEMS switch is developed and parameters are extracted based on the measured results. Using the electrical equivalent model, a high-isolation MEMS switch is designed and optimized by comparing three different series-shunt configurations, and then a very high isolation switch using 3-port ohmic MEMS switches has been developed where each MEMS switch is closely located. Thus the chip size is well compact to 1 mm × 1.2 mm. For both the 3-port ohmic switch and the series-shunt switch, the agreement of the measured and modeled RF performance demonstrates that the proposed electrical model can be used for evaluating the performance of RF MEMS systems.

## Figures and Tables

**Table 1.** Summary of mechanical design parameters.

**Properties**	**Simulated and calculated results**
Resonant frequency, *f*_0_ (KHz)	8.599338
Generalized mass, *m* (ng)	0.8118015
Pull-in voltage, *V_P_* (V)	19.5
Spring constant, *k* (N/m)	2.37
Reaction force, *F_r_* (μN)	≈ 7
Switch time, *t_on_* (μs)	48.5

**Table 2.** Extracted parameters of the 3-port MEMS switch.

**Parameters**	**Extracted values**
*R_on_* (Ω)	0.6
*L_on_* (pH)	195
*C_up_* (fF)	6.4
*C_g_* (fF)	10
*C_fon_* (fF)	8
*C_foff_* (fF)	10
